# Corneal power changes with Scheimpflug rotating camera after hyperopic LASIK

**DOI:** 10.1097/MD.0000000000013306

**Published:** 2018-12-14

**Authors:** Woong-Joo Whang, Young-Sik Yoo, Choun-Ki Joo

**Affiliations:** a Department of Ophthalmology and Visual Science, Yeouido St. Mary's Hospital; b Department of Ophthalmology and Visual Science, Seoul St. Mary's Hospital, College of Medicine, The Catholic University of Korea, Seoul, South Korea.

**Keywords:** corneal power, hyperopic LASIK, scheimpflug

## Abstract

To evaluate surgically induced refractive change (SIRC) by manifest refraction and corneal power changes using an automated keratometer and Scheimpflug rotating camera, and to find the best keratometric measurements reflecting SIRC after hyperopic laser-assisted in situ keratomileusis (LASIK).

This retrospective study included 18 eyes of 18 patients undergoing hyperopic LASIK using the Schwind Amaris 750S excimer laser. All measurements were performed preoperatively and 12 months postoperatively. Cycloplegic manifest refractions were performed and keratometric measurements were obtained via an RK-5 automated keratometer and a Pentacam rotating Scheimpflug camera. Sim K, true net power (TNP), and total corneal refractive power (TCRP) at 2.0 to 5.0 mm were analyzed using a Scheimpflug camera.

The mean manifest refractive changes in the spherical equivalent (SE) at the corneal plane were 2.32 ± 1.65 D at 12 months postoperatively. The refractive power changes by the automated keratometer and Sim K were significantly less than SIRC (*P* = .043 and *P* = .048, respectively). Both TNP and the TCRP in the 5.0 mm zone produced lesser mean differences with SIRC (0.05 D and 0.06 D) and showed closer agreements with SIRC on Bland-Altman plots and higher correlation coefficients with SIRC.

Corneal power measured on the anterior corneal surface underestimated SIRC. TCRP at the 5.0 mm zone provided by a Pentacam Scheimpflug camera reflected the SIRC accurately and precisely, and would be applicable for prediction of intraocular power before cataract surgery and follow-up measurement of corneal refractive power.

## 1 Introduction

Correction of hyperopia is a challenge for refractive surgeons, and several corrective methods have been introduced recently. Clear lens extraction is not an accurate or predictable method for hyperopic correction, and concerns have been raised about the loss of accommodation and postoperative complications, including macular edema and retinal detachment.^[1]^ Phakic intraocular lens (IOL) implantation can also cause postoperative complications.^[2,3]^

A previous study demonstrated that eyes undergoing hyperopic excimer laser surgery showed greater variability in corneal curvature and power than eyes treated with myopic excimer laser surgery.^[4]^ However, because the main site of ablation is not the central stroma but the mid-periphery of the cornea, impairment of the transparency of the axial cornea is not an apparent and significant disturbance of corrected distance visual acuity is expected to be unusual in hyperopic excimer laser surgery.

Consequently, hyperopic photorefractive keratectomy (PRK) and laser-assisted in situ keratomileusis (LASIK) are suggested as good options for correction of mild to moderate hyperopia.^[1,5]^ In a recent study, laser epithelial keratomileusis and transepithelial PRK also provided reasonable hyperopic correction outcomes.^[6,7]^ Wagh et al^[5]^ monitored the postoperative status of patients treated for hyperopic PRK over 18 years and found that refractive outcomes were stable between 7.5 years and 18 years after surgery. In a comparison of the flap creation methods used in hyperopic LASIK, femtosecond laser produced better results than a microkeratome.^[8,9]^

Accurate assessment of corneal power after hyperopic excimer laser surgery is very important. Finding the keratometric measurement that best reflects the surgically induced refractive change (SIRC) would be useful for both IOL power calculation and follow-up after refractive surgery.^[10]^ Several studies have investigated changes in corneal power after myopic excimer laser surgery and compared them with the SIRC. Corneal power changes at the 4.0 mm zone of the total optical power map accurately reflected SIRC in corneal topography.^[11,12]^ Corneal power at the 3.0 mm zone calculated by the Gaussian optics formula has been shown to reflect closely the refractive change on optical coherence tomography.^[13]^ Savini et al^[14]^ investigated changes in corneal power using a Scheimpflug rotating camera and demonstrated that total corneal refractive power (TCRP) at the 3.0 mm zone and 2.0 mm ring reflected SIRC accurately after myopic excimer laser surgery performed using the Allegretto Wave Eye-Q excimer laser (Wavelight Laser Technologie AG, Erlangen, Germany). On the other hand, Oh et al^[15]^ concluded that TCRP at the 4.0 mm zone produced the best results after wavefront-guided myopic PRK using the Visx STAR S4 IR CustomVue (AMO/Visx, Santa Ana, CA, USA). In a study using the dual Scheimpflug-Placido topographer, mean pupil power at 5.0 mm and 5.5 mm was the best option for reflecting postoperative corneal power calculated by clinical history.^[16]^ Although Gyldenkerne et al^[17]^ evaluated corneal power changes after hyperopic LASIK, they included myopic patients in spherical equivalent (SE).

However, there has been no study finding the best measurments reflecting SIRC after hyperopic LASIK at 1year postoperatively. The purpose of this study was to evaluate SIRC by manifest refraction and corneal power changes using an automated keratometer and Scheimpflug rotating camera and to find the keratometric measurement that best reflects SIRC after hyperopic LASIK.

## 2 Methods

Informed consent was obtained from all patients prior to the commencement of the study, and the methods used adhered to the tenets of the Declaration of Helsinki for use of human participants in biomedical research. The study was approved by the institutional review board of Seoul St. Mary's Hospital.

This retrospective study included 18 eyes (8 right eyes; 10 left eyes) of 18 patients undergoing hyperopic excimer laser surgery at Seoul St. Mary Hospital between March 2014 and April 2015. All patients discontinued wear of contact lenses for more than 4 weeks before their surgery.

Inclusion criteria were hyperopia that had been stable for at least 12 months prior to surgery and corrected distance visual acuity of more than 20/32 (0.2 logMAR) preoperatively. Lenticular changes can affect the refractive outcome, so patients with any sign of cataract were excluded and only patients under 40 years of age were included. Patients with high astigmatism (>3.00 D on even one measurement), previous ocular surgery or trauma, corneal disease including keratoconus, glaucoma, macular disease, peripheral retinal degeneration, active ocular or systemic disease, amblyopia, and pregnancy were also excluded.

All surgical procedures were performed by an experienced surgeon (CKJ). The corneal flap was created with the IntraLase femtosecond laser (AMO) using a superior hinge, a diameter of 9.5 mm, and flap thickness of 100 μm. Hyperopic excimer laser surgery was performed using the Schwind Amaris 750S excimer laser (Schwind eye-tech-solutions, GmbH & Co. KG, Kleinostheim, Germany) with an optical zone of 7.0 mm. All measurements were performed preoperatively and 12 months postoperatively.

Cycloplegic manifest refractions were performed and keratometric measurements were obtained using an RK-5 automated keratometer (Canon Inc., Tokyo, Japan) and a Pentacam rotating Scheimpflug camera (Oculus, Wetzler, Germany). A Pentacam HR (Oculus) was used to analyze the cornea via 25-picture scanning, and only scans that had an examination-quality specification graded by the instrument as “OK” were included in the study. The mean value from 5 times of measurement was applied and the measurements were performed by skilled experts (CR: WJW; automated keratometry and Scheimpflug rotating camera: YSY). The following corneal measurements were analyzed using the Pentacam Scheimpflug rotating camera both preoperatively and postoperatively:

Sim K – this value is the arithmetic mean of a pair of meridians spaced 90 degrees apart, and with the greatest difference in axial power lying within a central 3.0 mm zone. It is calculated by entering the corneal curvature radius into a thin-lens formula for paraxial imagery, which considers the cornea as a single refractive sphere. The corneal radii are converted into dioptric power values using the keratometric index of refraction (1.3375).

True net power – this value is calculated using the Gaussian optics formula.

Total corneal refractive power – TCRP is automatically measured by the ray tracing method and is calculated using the values for the anterior radius, posterior radius, and corneal thickness. Snell's law and the specific refractive indices of air, the cornea, and the aqueous humor are used to calculate the corneal power. TCRPs were measured at the 2.0 to 5.0 mm zones and 2.0 to 5.0 mm rings (zone, corneal power measured over the inner zone; ring, corneal power measured over a ring). Due to centration, TCRP can be measured in two ways, that is, centered on the apex and centered on the pupil. We analyzed corneal powers centered on the apex because of the pupil center shifts with changes in pupil size. From these corneal powers, we analyzed the flattest keratometric value (flat K), steepest keratometric value (steep K), mean keratometric value (mean K), and astigmatism. There was no development of cataract or complications such as corneal haziness during 12 months of follow-up.

SIRC was defined by subtracting the postoperative SE from the preoperative SE by manifest refraction and corneal dioptric power change was calculated by subtracting the preoperative corneal power from the postoperative corneal power. Difference with SIRC was defined as a value obtained by subtracting the SIRC from corneal dioptric power change. We also evaluated the absolute value of difference between SIRC and corneal dioptric power change.

The statistical analysis was performed using Statistical Package for the Social Sciences version 21.0 software (IBM Corp., Armonk, NY, USA). The values did not show a Gaussian distribution, so refractive changes were compared using the Friedman test with Dunn's post test. The Wilcoxon signed rank test was used to compare the preoperative refractive power with the postoperative refractive power. Spearman's correlation test was used to analyze the correlation between change in each corneal power and SIRC. Bland-Altman plots and linear regression analysis were also used to evaluate the agreement between corneal power change and SIRC. A *P*-value ≤.05 was considered to be statistically significant.

## 3 Results

**Figure 1:**
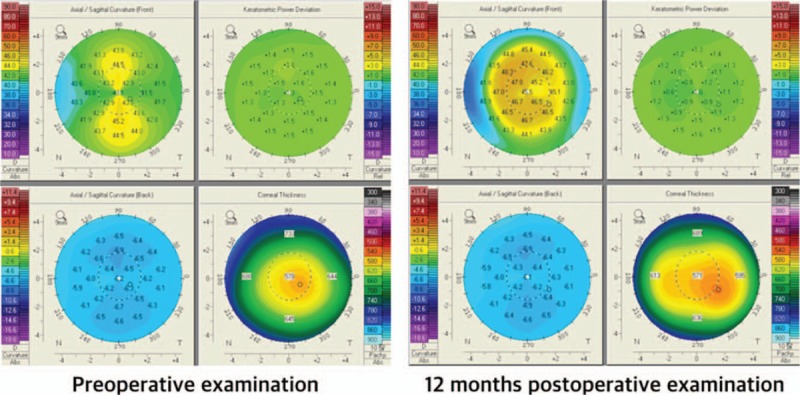
An example of one patient's preoperative and 12 months postoperative data. (Eye #4).

Table 1 shows demographic data in 18 eyes and Figure 1 shows an example of 1 patient's preoperative and 12 months postoperative data on Scheimpflug camera. The axial map shows the steepening of central cornea and the corneal thickness map shows decrese of peripheral corneal thickness. Table 2 shows the preoperative and postoperative corneal refractive powers measured by the automated keratometer and Scheimpflug rotating camera. All corneal powers showed statistically significant changes after hyperopic LASIK (all *P* < .001)

**Table 1:**
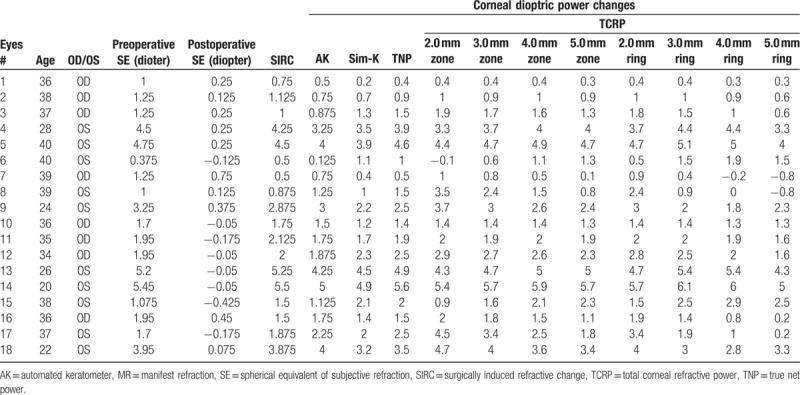
Descriptive preoperative and postoperative data measured by subjective refraction, automated keratometer, and Scheimpflug rotating camera in 18 eyes.

**Table 2:**
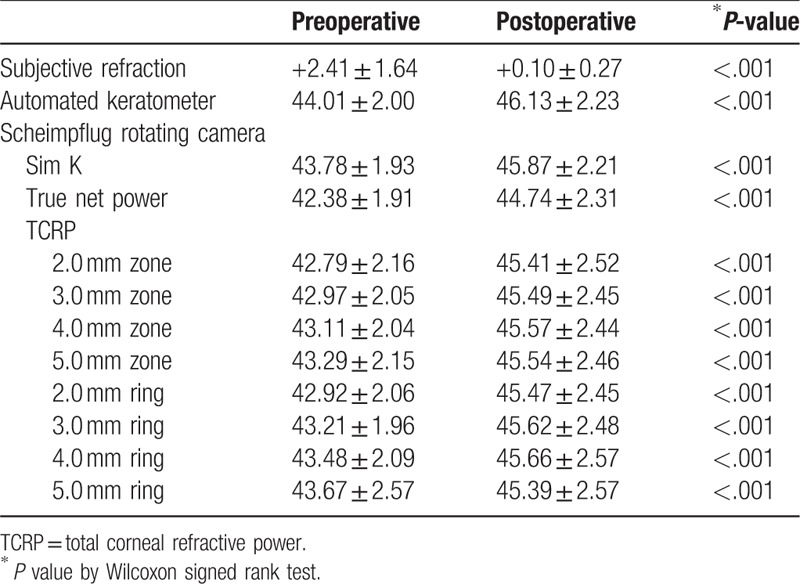
Preoperative and postoperative data measured by subjective refraction, automated keratometer, and Scheimpflug rotating camera.

The changes in subjective refraction and corneal powers are shown in Table 3. The mean manifest refractive change in SE at the corneal plane was 2.32 ± 1.65 D by 12 months postoperatively, and the Friedman test showed statistically significant differences between the refractive changes (*P* < .001). The refractive power changes measured by the automated keratometer were significantly different from the SIRC (*P* = .043). In the Scheimpflug camera analysis, the changes in Sim K and TCRP at the 5.0 mm ring were also significantly different from the SIRC (*P* = .048 and *P* < .001, respectively). The absolute values of the differences from SIRC were the lowest for change in true net power (TNP) and followed by TCRP at the 5.0 mm zone, 3.0 mm ring, 4.0 mm zone, and 4.0 mm ring (0.05, 0.06, 0.09, 0.14, and 0.14 D, respectively). On the Bland-Altman plots, the change in TCRP at the 4.0 mm zone showed the closest agreement with SIRC, followed by TCRP at the 5.0 mm zone, automated K, TNP, and Sim K. The mean absolute values from the differences between SIRC and corneal dioptric power changes were the lowest for TCRP at the 5.0 mm zone (0.34D) and followed by TCRP 4.0 mm zone, automated keratometer, and TNP. Table 4 shows mean differences with SIRC and mean absolute values from differences with SIRC in 2 subgroups.

**Table 3:**
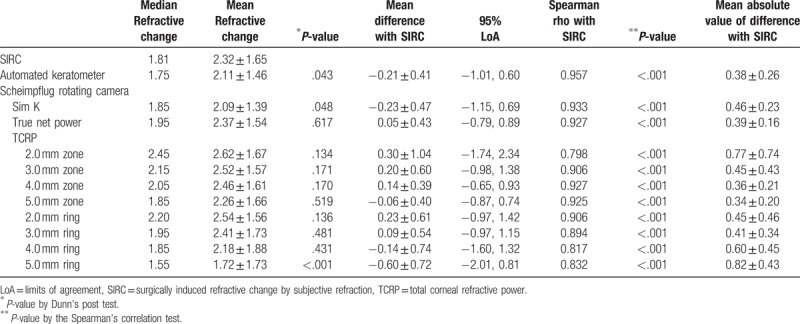
SIRC measured by manifest refraction and corneal power changes by automated keratometer and Scheimpflug rotating camera.

**Table 4:**
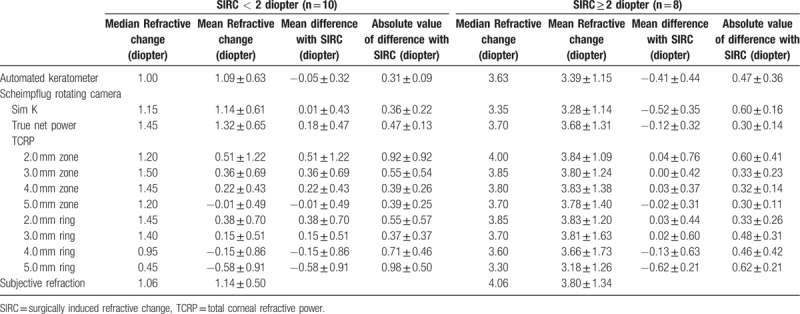
Refractive changes measured by manifest refraction (SIRC), mean differences with SIRC, and mean absolute values from differences with SIRC in 2 subgroups.

**Figure 2:**
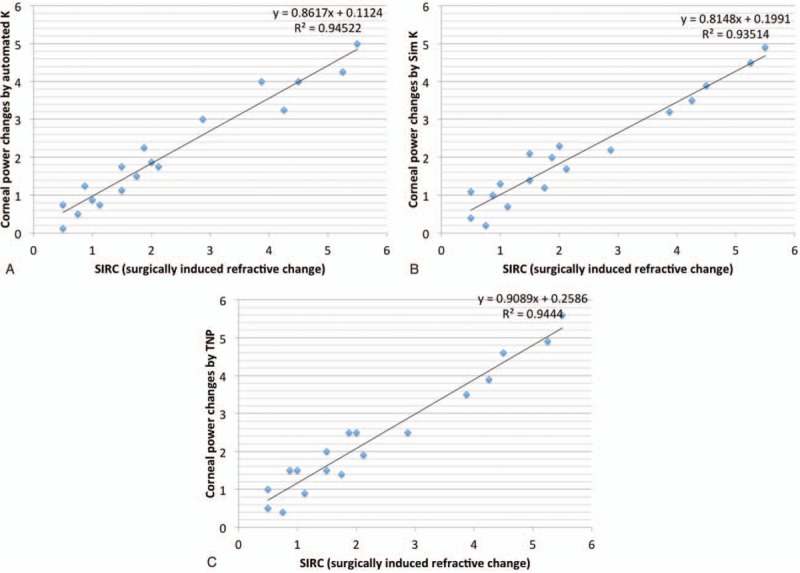
Scatter diagram showing the correlation between the SIRC and the refractive changes indicated by each measurement. (A) Corneal refractive power by the automated keratometer, (B) simulated K determined by the Scheimpflug rotating camera, and (C) TNP calculated by the Scheimpflug rotating camera. SIRC = surgically induced refractive change, TNP = true net power.

Figures 2 and 3 are scatter diagrams demonstrating the differences between changes in corneal power and the SIRC. All the corneal power changes showed a statistically significant correlation with SIRC, and the change measured by automated keratometer showed the highest correlation, followed by Sim K, TNP and TCRP at the 4.0 mm and 5.0 mm zones (*rho* = 0.957, 0.933, 0.927, 0.927, and 0.925, respectively).

**Figure 3:**
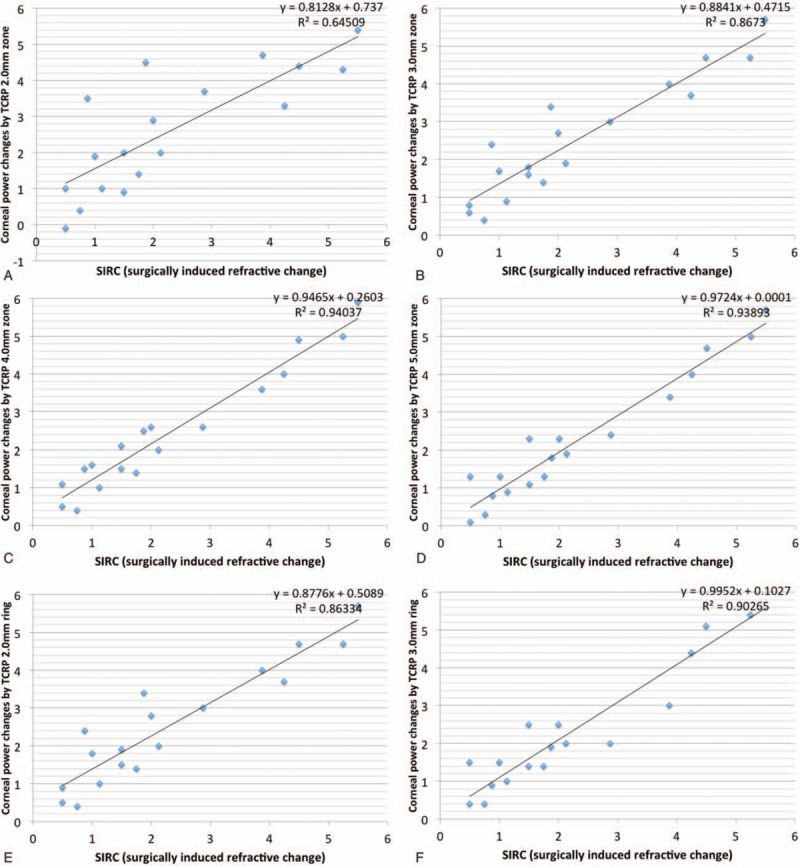
Scatter diagram showing the correlation between the SIRC and the changes in TCRP provided by the Scheimpflug rotating camera. (A) 2.0 mm zone, (B) 3.0 mm zone, (C) 4.0 mm zone, (D) 5.0 mm zone (E) 2.0 mm ring, (F) 3.0 mm ring, (G) 4.0 mm ring, and (H) 5.0 mm ring. SIRC = surgically induced refractive change, TCRP = total corneal refractive power.

**Figure 3 (Continued):**
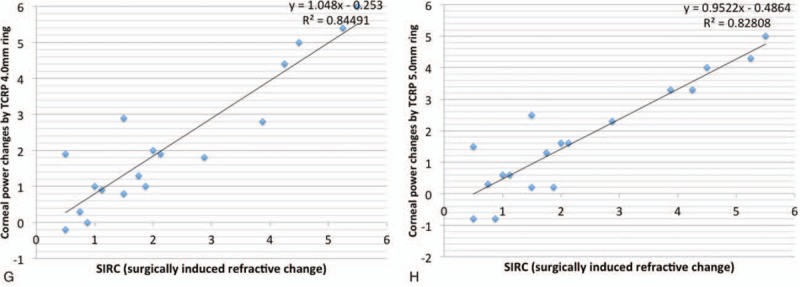
Scatter diagram showing the correlation between the SIRC and the changes in TCRP provided by the Scheimpflug rotating camera. (A) 2.0 mm zone, (B) 3.0 mm zone, (C) 4.0 mm zone, (D) 5.0 mm zone (E) 2.0 mm ring, (F) 3.0 mm ring, (G) 4.0 mm ring, and (H) 5.0 mm ring. SIRC = surgically induced refractive change, TCRP = total corneal refractive power.

## 4 Discussion

Corneal power measured only at the anterior corneal surface underestimates SIRC after hyperopic excimer laser surgery. Total corneal power measurements produce better results. Automated keratometer data and data derived from Sim K obtained via a rotating Scheimpflug camera indicate less change in dioptric power than with SIRC, and these results may be attributable to the keratometric index problem. A refractive index of 1.3375 is established when the corneal thickness is constant at 600 μm.^[18]^ As the thickness increased toward the periphery in the normal cornea, a corrected refractive index of 1.3273 to 1.3315 was introduced in recent studies.^[19]^ The eye with a history of hyperopic excimer laser surgery has a relatively small difference in corneal thickness between the center and periphery, and a greater posterior/anterior instantaneous radii of curvature ratio, (0.86 versus 0.82)^[20]^ and a greater the refractive index comparing with the normal cornea.^[21,22]^ Consequently, the keratometric values for the anterior cornea overestimated the preoperative corneal power and consequently underestimated the change in corneal power. Previous studies have reported similar results. Wang et al^[21]^ evaluated 2 types of corneal topography, that is, the EyeSys (EyeSys Technologies, Houston, TX, USA) and the Humphrey Atlas (Carl Zeiss Meditec, Jena, Germany), and concluded that both methods underestimated the actual corneal power changes at follow-up 3 months later. Rosa et al^[23]^ also found that corneal power changes measured by the automated IOLMaster keratometer (Carl Zeiss Meditec) underestimated the SIRC at 6 months postoperatively.

As a means of solving the keratometric index problem, the Pentacam Scheimpflug camera provides TNP and equivalent K readings via the Gaussian optics formula. The corneal power changes derived from TNP, the TCRP 3.0 mm ring, and the TCRP 5.0 mm zone in particular are less different from the SIRC. Among the above parameters, the TNP and the TCRP 5.0 mm zone showed higher correlation coefficients and narrower 95% limits of agreement on Bland–Altman plots. This result is different from the result of previous study investigating corneal power changes after hyperopic LASIK. Glydenkern et al^[17]^ concluded that 2.0 mm ring centered on apex reflected SIRC best and these contrasting results supposed to be caused by study population. We only included preoperatively hyperopic patients on SE.

Wang et al^[20]^ compared the Gaussian equivalent power calculated by the Gaussian optics formula with TCP by the ray tracing method at the 4.0 mm zone using the dual Scheimpflug analyzer and concluded that Gaussian equivalent power tended to be more than TCP in normal eyes but the absolute differences between the 2 corneal powers decreased in hyperopic LASIK/PRK eyes. However, TNP calculated by the Gaussian optics formula had the lowest value preoperatively and postoperatively, and this is consistent with previous studies using the Scheimpflug rotating camera.^[19,24]^ TNP showed disappointing results in studies investigating changes after myopic excimer laser surgery; changes in TNP underestimated SIRC, with differences ranging from 0.25 D to 0.74 D.^[14,15]^ In contrast, TNP was a good indicator for reflecting manifest refraction, and the mean difference was 0.05 D in our study. This finding is not consistent with previous studies investigating corneas with myopic excimer laser surgery and is thought to be caused by a different ratio for the posterior/anterior instantaneous radii of curvature.^[20]^ Although the change in TNP was the least different from the SIRC, TNP did not seem to reflect SIRC as much as the TCRP 5.0 mm zone. From the regression analysis in Figure 2C and Figure 3 D, the change in TNP was found to be greater than the SIRC in the correction of lower hyperopia and the TNP change tended to underestimate the SIRC in high-degree correction (TNP change = 0.9089 × SIRC + 0.2586, TCRP 5.0 mm zone change = 0.9724 × SIRC + 0.0001). This result suggests that the correspondence between TNP change and SIRC could be reduced if the study population changed. Refractive surgeons need to be more cautious in the use of TNP. Table 4 shows mean differences with SIRC, and mean absolute values from differences with SIRC in 2 subgroups classified by the amount of SIRC. Corneal power change by TNP was greater than SIRC when SIRC was less than 2 diopter. When the SIRC is relatively large, corneal power change by the TNP tends to decrease. On the other hand, the corneal dioptric power changes in TCRP 5.0 mm zone were less than 0.1 diopters in both subgroups.

TCRP is calculated by the ray tracing method based on Snell's law, and another difference between TNP and TCRP is the reference used. TCRP is referenced to the anterior surface of the cornea while TNP is referenced to the second principal plane in front of the cornea. The normal cornea has a positive spherical aberration, so the TCRP zone and ring increased with the measurement area preoperatively. This is consistent with a previous report for the normal cornea.^[19]^ However, the postoperative cornea shows a different pattern. The positive spherical aberration changes to a negative one and corneas become more prolate after hyperopic excimer laser surgery.^[25,26]^ Corneal refractive powers at TCRP 5.0 mm were less than those at TCRP 4.0 mm in terms of both zone and ring. As the measurement area increased, the absolute value of corneal power changes decreased. This result was the opposite to some previous findings after myopic corneal refractive surgery.^[14,15]^ However, it was consistent with a prior study evaluating eyes with a history of hyperopic excimer laser surgery using a Keratron Scout videokeratoscope (Optikon, Rome, Italy).^[27]^

Unlike previous studies demonstrating the accuracy of TCRP in the 3.0 to 4.0 mm zone after myopic excimer laser surgery, TCRP in the 5.0 mm zone produced the results that best reflected SIRC. This could be caused by the optical zone. Because the main site of ablation in hyperopic excimer laser surgery is the mid-periphery of the cornea and hyperopic correction provides a smaller postoperative functional optical zone,^[28]^ the optical zone planned for hyperopic excimer laser surgery tends to be larger than for myopic excimer laser surgery. We performed ablation of the 7.0 mm optical zone in all our patients. In a comparison of eyes undergoing small incision lenticule extraction due to the lenticular diameter, the TCRP 2.0 mm ring performed best with a lenticular diameter of 6.2 mm and the TCRP 5.0 mm ring performed best with a lenticular diameter of 6.5 mm.^[29]^

There are some limitations to the present study. Firstly, it did not include high hyperopia (SE in the range of +0.38 D to +4.75D, mean +2.41 D). Although some studies define the limit of hyperopic laser vision correction as +3.00 D to +4.00 D, an analysis investigating corneal power changes with high hyperopia would be helpful in the future. Secondly, the optical zone of hyperopic correction was 7.0 mm in all cases. Given that some studies have demonstrated that an optical ablation zone of 6.0–6.5 mm induces less high-order aberrations,^[27,30]^ an investigation of corneal power changes with a smaller optical zone ablation is needed.

In conclusion, corneal powers on the anterior corneal surface underestimate the SIRC. TCRP at the 5.0 mm zone provided by a Scheimpflug camera reflects the SIRC accurately and precisely, and may be useful for prediction of intraocular power before cataract surgery and follow-up measurement of corneal refractive power.

## Author contributions

**Conceptualization:** Woong-Joo Whang and Choun-ki Joo.

**Data curation:** Woong-Joo Whang.

**Formal analysis:** Woong-Joo Whang and Young-Sik Yoo.

**Investigation:** Woong-Joo Whang.

**Methodology:** Woong-Joo Whang, Young-Sik Yoo, and Choun-ki Joo.

**Project administration:** Young-Sik Yoo.

**Writing – original draft:** Woong-Joo Whang.
